# Changes in the Secondary Structure and Assembly of Proteins on Fluoride Ceramic (CeF_3_) Nanoparticle Surfaces

**DOI:** 10.1021/acsabm.2c00239

**Published:** 2022-06-02

**Authors:** Naoya Sakaguchi, Samal Kaumbekova, Ryodai Itano, Mehdi Amouei Torkmahalleh, Dhawal Shah, Masakazu Umezawa

**Affiliations:** † Department of Materials Science and Technology, Faculty of Advanced Engineering, Tokyo University of Science, 6-3-1 Niijuku, Katsushika, Tokyo 125-8585, Japan; ‡ Department of Chemical and Materials Engineering, School of Engineering and Digital Sciences, Nazarbayev University, Kabanbay Batyr 53, Nur-Sultan 010000, Kazakhstan

**Keywords:** amyloid β peptide, fluoride nanoparticles, infrared spectrometry, molecular dynamics, β-sheet

## Abstract

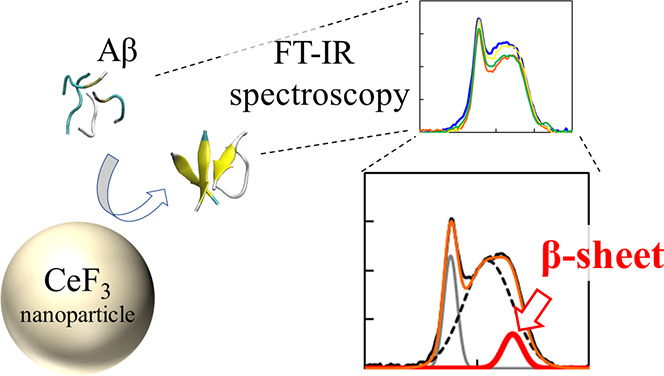

Fluoride nanoparticles (NPs) are materials utilized in the biomedical field for applications including imaging of the brain. Their interactions with biological systems and molecules are being investigated, but the mechanism underlying these interactions remains unclear. We focused on possible changes in the secondary structure and aggregation state of proteins on the surface of NPs and investigated the principle underlying the changes using the amyloid β peptide (Aβ_16–20_) based on infrared spectrometry. CeF_3_ NPs (diameter 80 nm) were synthesized via thermal decomposition. Infrared spectrometry showed that the presence of CeF_3_ NPs promotes the formation of the β-sheet structure of Aβ_16–20_. This phenomenon was attributed to the hydrophobic interaction between NPs and Aβ peptides in aqueous environments, which causes the Aβ peptides to approach each other on the NP surface and form ordered hydrogen bonds. Because of the coexisting salts on the secondary structure and assembly of Aβ peptides, the formation of the β-sheet structure of Aβ peptides on the NP surface was suppressed in the presence of NH_4_^+^ and NO_3_^–^ ions, suggesting the possibility that Aβ peptides were adsorbed and bound to the NP surface. The formation of the β-sheet structure of Aβ peptides was promoted in the presence of NH_4_^+^, whereas it was suppressed in the presence of NO_3_^–^ because of the electrostatic interaction between the lysine residue of the Aβ peptide and the ions. Our findings will contribute to comparative studies on the effect of different NPs with different physicochemical properties on the molecular state of proteins.

## Introduction

In the field of material sciences, the self-assembly of molecules is a major area of interest for researchers to develop soft matter.^1^ Furthermore, in biology, the secondary structure and assembly state of a protein are important for regulating the function and activity of the protein. The formation of fibrillar aggregates of peptides and proteins is associated with various diseases including neurogenerative disorders.^2^ The dysregulation of the structure and state of proteins can lead to diseases in biological organs.^3,4^ For example, fibril formation via the self-assembly of denatured proteins causes amyloid diseases, such as Alzheimer’s disease,^5^ which is the most common neurodegenerative disease and is associated with cognitive and physical decline.^6^ Pathologically, in patients with Alzheimer’s diseases, senile (neuritic) plaques of the amyloid beta (Aβ) protein are observed in the brain tissue.^7^

Amino acid residues in proteins interact with each other through hydrogen bonds, disulfide bonds, electrostatic interactions, and hydrophobic interactions; these interactions affect protein conformation. As the distance between peptides reduces due to protein aggregation, their secondary structure can change due to an increase in interactions among peptides, for example, an increase in the β-sheet structure via enhanced hydrogen bonds. Although Aβ generally aggregates more at higher concentrations, even at low concentrations, it forms a β-sheet structure that can promote self-assembly via aromatic interactions between phenylalanine residues.^8,9^ Especially, the hydrophobic domain of Aβ—the region around residues 17–20, LVFF—is important for β-sheet formation.^10^

In general, changes in the secondary structure and protein aggregation can be enhanced at liquid–liquid and liquid–air interfaces, as observed through denaturation by surfactants. In addition to these interfaces, liquid–solid interfaces are also likely to be the sites for protein denaturation because the domains with high affinity for the solid are exposed to the molecular surface. Molecular dynamics (MD) simulations have suggested that the structural arrangement of Aβ attached to the surface of carbon nanotubes—an inorganic nanomaterial—changes with the radius of the nanotubes.^11^ The rate of peptide aggregation on the solid–liquid interface is determined by the affinity (binding force) between the peptide and the solid material and the shape (roughness) of the solid surface.^12^ While a high affinity between peptides and solid surfaces enhances their adsorption and inhibits the self-assembly (aggregation) of peptides, solid materials with middle affinities and a rough surface (with a nanoscale morphology) accelerate the aggregation of peptides. In contrast, in a study in which all-atom MD simulations of the conformation of Aβ_16–22_ peptides were performed, hydrophobic interactions were found to prevent the formation of β-sheet structures in the presence of gold nanoparticles (NPs).^13^ The charge on the surface of NPs is also likely to be important for Aβ fibrillation. Negatively charged NPs inhibit the formation of Aβ fibrillation, whereas positively charged NPs have no effect on fibril formation.^14^ The aggregation dynamics of Aβ peptides (Aβ_16–21_) on fullerene NP models was also investigated using MD simulation along with the effect of the coexistence of ionic salts.^15^ However, there has still been no experimental evidence of how Aβ peptides behave on NP surfaces in physiological environments containing salts.

In the present study, fluoride ceramic NPs, which are expected to be applied to the biomedical field, were used as a target material. Fluoride has a moderate phonon energy of 350 cm^–1^ and has been widely utilized in the biomedical field for applications such as brain imaging^16−18^ as a fluorescent contrast agent containing rare-earth ions.^19−22^ This is because it has both high chemical durability, as a lower phonon energy reduces the chemical stability similarly to chlorides, and high luminescence efficiency when doped with rare earths because higher phonon energy causes quenching via enhanced thermal relaxation.^23^ Fluoride nanomaterials labeled within the long-wavelength (>1000 nm) near-infrared (NIR) region, called the second and third NIR (NIR-II/III) biological windows,^24^ have been developed for NIR fluorescence computed tomography,^25^ photodynamic therapy,^26^ and fluorescence nanothermometers^27,28^ for in vivo investigations of deep tissues such as tissues in the peritoneal cavity.^29^ Rare-earth-doped fluoride crystals have also been developed for application in lifetime-based NIR fluorescence thermometers.^30^ Fluoride crystals containing Gd^3+^ (e.g., NaGdF_4_) and luminescent rare earths have been developed for bimodal imaging in fluorescence and magnetic resonance modalities.^31−36^ CeF_3_ NPs were used in this study as a fluoride ceramic that can show a surface reactivity similar to that of the fluorides, as mentioned above. The aim of this study was to investigate the effect of fluoride ceramic NPs, which are expected to have further biomedical applications, on the conformation and assembly of Aβ molecules using an in vitro experimental system and MD simulation.

## Results and Discussion

**Figure 1:**
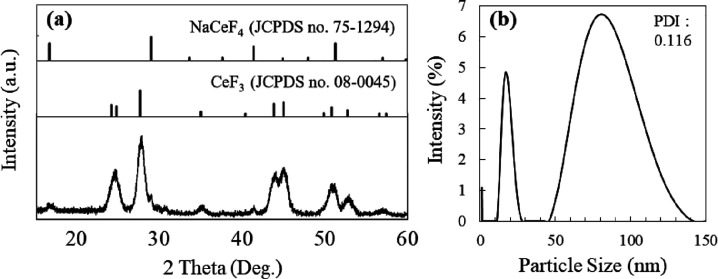
Characterization of fluoride NPs used in this study. (a) XRD pattern of the samples and references of CeF_3_ (JCPDS no. 08-0045) and NaCeF_4_ (JCPSD no. 75-1294). (b) DLS spectra showing the hydrodynamic diameter of the fluoride NPs dispersed in cyclohexane.

Fluoride NPs synthesized in this study were characterized using X-ray diffraction (XRD) and dynamic light scattering (DLS). As shown in Figure 1a, the XRD patterns showed that the NPs were majorly CeF_3_ with small amounts of NaCeF_4_. Data from DLS showed that the CeF_3_ NPs showed a major peak at a diameter of 80 nm with a low polydispersity index (0.116), although it contained a minor fraction peak at 20 nm. We considered the representative CeF_3_ particle size to be 80 nm of the peak in the size distribution in our following investigations.

**Figure 2:**
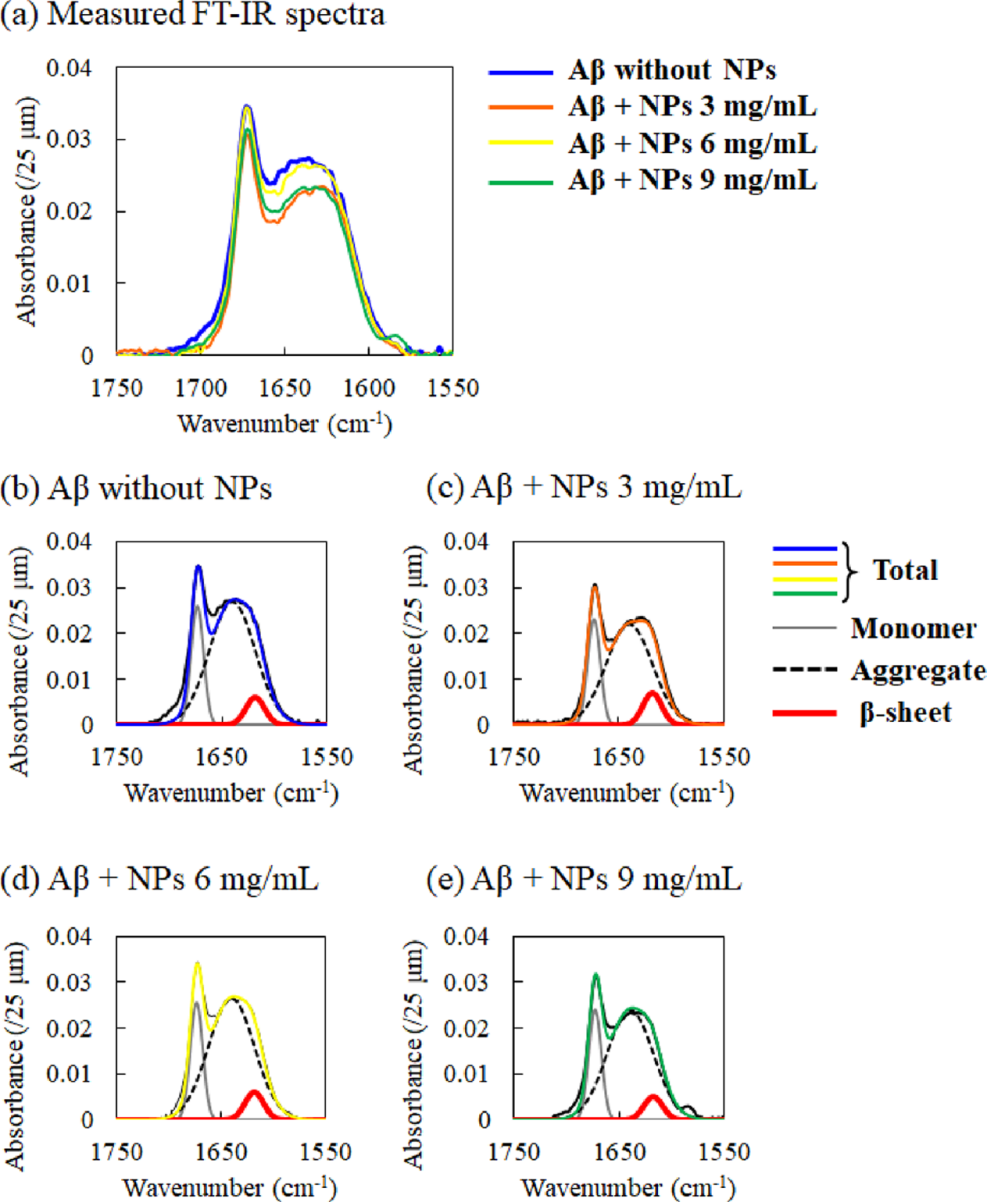
Amide I band in the FT-IR spectra of Aβ_16–20_ interacted with CeF_3_ NPs. (a) FT-IR spectra of Aβ_16–20_ (6 mg/mL) that interacted with different concentrations of CeF_3_ NPs (3, 6, and 9 mg/mL). (b–e) Deconvolution results via Gaussian fitting for the amide I band in FT-IR spectra of Aβ_16–20_ (6 mg/mL) with CeF_3_ NP concentrations of (b) 0, (c) 3, (d) 6, and (e) 9 mg/mL.

In this study, fragment peptides with small molecular weights, Aβ_16–20_, which allow MD simulations to be performed easily, were used to investigate the secondary conformational changes and aggregation of Aβ using both Fourier transform infrared (FT-IR) spectroscopy and the MD simulations. CeF_3_ NPs were dispersed in an aqueous solution to interact with Aβ_16–20_ (KLVFF) in the aqueous solution. FT-IR spectroscopy was used for analyzing the secondary structure of proteins. Especially, the amide I vibration peak (appearing around 1650 cm^–1^ and mainly attributed to C=O stretching) was focused because it is hardly affected by the nature of the side chains but depends on the secondary structure of the backbone.^37^ Thus, it is commonly used for the secondary structure analysis^38^ that can also be applied to in situ analyses under microscopy.^39^ Not only the secondary structure but also the aggregation of the Aβ peptide in solvents can be analyzed using FT-IR spectroscopy.^40^ Hydrochloric acid (1 mmol/L) was used to maintain the dispersibility of the CeF_3_ NPs. However, the water molecule showed peaks not only at 3300 cm^–1^ but also at 1650 cm^–1^ in the IR region; these peaks interfere with those of the amide I band at 1650 cm^–1^,^9^ which is the target of analysis in this study. Therefore, deuterium chloride and deuterium oxide were used, instead of hydrochloric acid and water, as the dispersion media for CeF_3_ and solution of Aβ_16–20_. Deuterium chloride did not affect the FT-IR spectra of Aβ_16–20_ solution at this concentration (final 0.25 mmol/L) (data not shown). FT-IR spectra of the samples in which Aβ_16–20_ was interacted with different concentrations of CeF_3_ NPs were analyzed. As shown in Figure 2a, Aβ_16–20_ showed two major peaks at 1674 and 1640 cm^–1^, which correspond to aggregates and monomers, respectively.^9^ Deconvolution analysis using Gaussian fitting showed that the FT-IR absorption spectra of Aβ_16–20_ also included, in addition to the major peaks, a minor peak at 1618 cm^–1^ corresponding to β-sheet formation of Aβ_16–20_^9,38^ (Figure 2b). The β-sheet formation of Aβ_16–20_ (6 mg/mL) increased in the presence of 3 mg/mL CeF_3_ NPs, and this increase was not observed in the presence of 6 mg/mL CeF_3_ NPs as the ratios of the β-sheet peak in the total amide I absorption were 6.8, 9.3, 6.9, and 6.3% in Aβ_16–20_ that interacted with 0, 3, 6, and 9 mg/mL of CeF_3_ NPs, respectively (Figure 2b–e and Table 1). This may be due to the difference in the number of Aβ molecules per surface area of the NPs, which is the site of the NP–protein interaction in the system. Because the shape of CeF_3_ NPs (density: 6.16 g/cm^3^) is approximated to be a sphere with a diameter of 80 nm (2.7 × 10^5^ nm^3^/particle), the mass and surface area are 1.7 × 10^–15^ g and 2.0 × 10^4^ nm,^2^ respectively, leading to a specific surface area per mass of 1.2 × 10^19^ nm^2^/g. The surface area of the CeF_3_ particles in a dispersion of 3 mg/mL was 3.7 × 10^16^ nm^2^/mL, while the total particle surface area in the dispersion was proportional to the particle concentration. Although the ratio of Aβ molecules that were attracted to the NP surface, that is, their local enrichment rate on the surface, in the dispersion was unknown, our results suggest that a certain enrichment of Aβ molecules promotes intermolecular bonding, thereby promoting β-sheet formation.

**Table 1:** Ratio of Each Component in the Amide I Band of the FT-IR Spectra of Aβ_16–20_ (6 mg/mL) That Interacted with Different Concentrations of CeF_3_ NPs^a^

	Aβ	Aβ + NPs 3 mg/mL	Aβ + NPs 6 mg/mL	Aβ + NPs 9 mg/mL
monomer	0.20	0.20	0.20	0.20
aggregate	0.74	0.70	0.74	0.73
β sheet	0.068	0.093	0.069	0.063

a The ratios were obtained via deconvolution of the amide I band observed in each sample.

**Figure 3:**
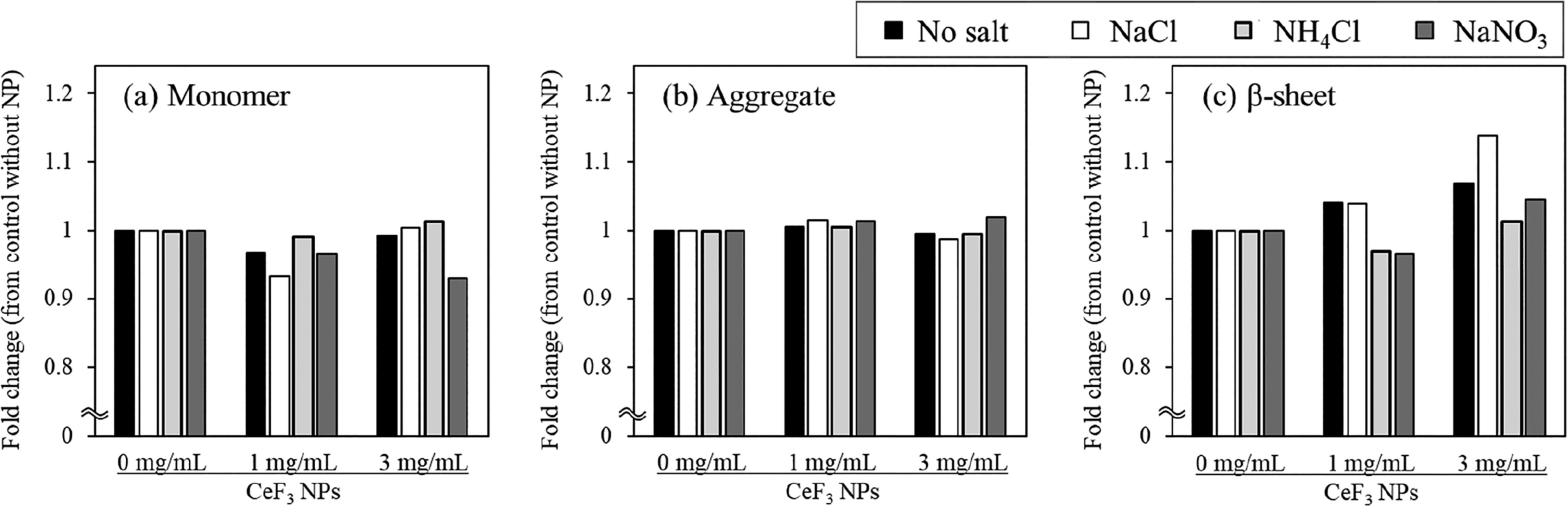
Increase in each separated peak (fold-change) in the amide I band of Aβ_16–20_ dissolved with salts due to CeF_3_ NPs. The fold changes of the peaks of the (a) monomer (1674 cm^–1^), (b) aggregate (1640 cm^–1^), and (c) β-sheet (1618 cm^–1^) of Aβ_16–20_ (6 mg/mL) dissolved in D_2_O containing each salt (0.15 M) due to the coexistence of CeF_3_ NPs (1 and 3 mg/mL) are shown.

**Figure 4:**
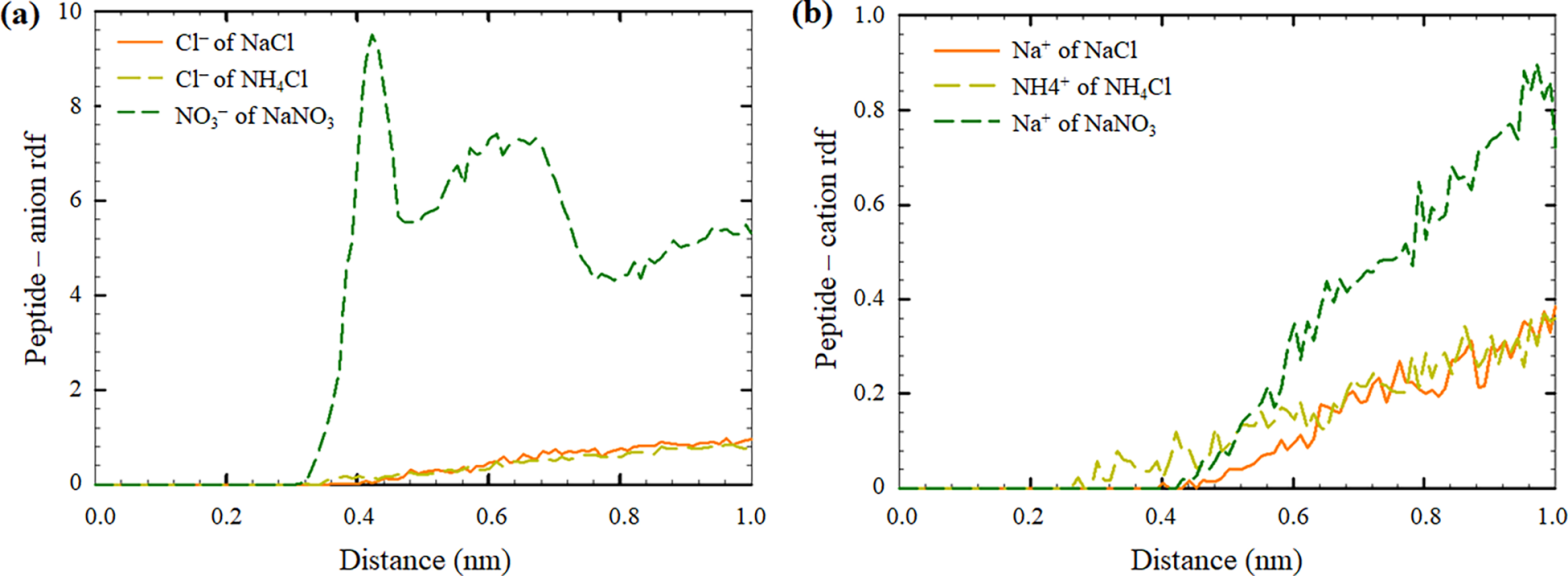
rdf plots of the interactions between Aβ_16–20_ peptides and the (a) anions and (b) cations. The results were averaged among four peptides present in the systems at the end of the MD simulations, when the systems were stabilized (last 10 ns of the MD run).

**Figure 5:**
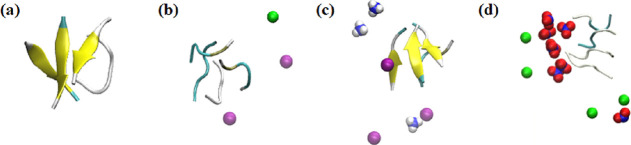
Representative snapshots of the peptide aggregates and ions within 0.1 nm of peptides in the systems under study: (a) no salt, (b) 0.15 M NaCl, (c) 0.15 M NH_4_Cl, and (d) 0.15 M NaNO_3_. Coloring methods in VMD: 1. Secondary structure of the peptide: beta sheet = yellow, beta bridge = tan, bend = cyan, turn = cyan, and coil = white. 2. Ions: Na^+^ = green, NH_4_^+^ = blue and white, Cl^–^ = purple, and NO_3_^–^ = blue and red.

The effect of coexisting ions on the behavior of the Aβ_16–20_ peptide on the surface of CeF_3_ NPs was studied using Aβ_16–20_ in D_2_O with dissolved NaCl, NH_4_Cl, and NaNO_3_ (0.15 M). The effects of these ions at the same concentration were investigated in this experiment to compare the principle of action of each ion. Even without CeF_3_ NPs, NH_4_^+^ promoted β-sheet formation of Aβ_16–20_, whereas NO_3_^–^ enhanced the monomer retention of Aβ_16–20_ (Table 2). Na^+^ and Cl^–^ did not affect the amide I band of Aβ_16–20_ solution in D_2_O (Table 2). β-Sheet formation of Aβ_16–20_ (6 mg/mL) was increased (14%) by CeF_3_ NPs (3 mg/mL) in the presence of NaCl as well as in the absence of salts, whereas no elevation of β-sheet formation by NPs was observed in the presence of NH_4_^+^ and NO_3_^–^ (Figure 3). The results suggest that NH_4_^+^ and NO_3_^–^ suppressed the β-sheet formation of Aβ promoted on CeF_3_. MD simulations were further performed on four monomers of Aβ_16–20_ in the presence of these salts. The findings showed that the NO_3_^–^ was strongly bound to the peptide as compared to chloride in the absence of NPs. The average distance between the peptide and NO_3_^–^ was 0.67 nm, whereas the distance with Cl^–^ was 0.99 nm (Table 3). Elevated peaks were observed on the radial distribution function (rdf) plots between peptide residues and NO_3_^–^ and Na^+^ of NaNO_3_ with maximum peak values of ∼9.5 at a 0.42 nm distance, as shown in Figure 4a, and ∼0.88 at a 0.97 nm distance, as shown in Figure 4b). In contrast, comparatively low peak values were observed between peptides and ions of NaCl and NH_4_Cl (with maximum peak values of ∼0.96 at a 0.95 nm distance, as shown in Figure 4a, and ∼0.38 at a 1.0 nm distance, as shown in Figure 4b), indicating their weak interactions. Among the different residues of Aβ_16–20_ (KLVFF), NO_3_^–^ strongly interacted with the lysine (K-16) residue, with the average distance between lysine and NO_3_^–^ being 0.4 nm (Table 3). The possible reasons for lysine and NO_3_^–^ interactions are as follows: 1) the strong electrostatic interactions between positively charged lysine and anions and 2) the formation of hydrogen bonds between lysine’s sidechain and NO_3_^–^.^41^ Consequently, these strong interactions suppressed the formation of β-sheets in the secondary structures of Aβ_16–20_ peptides in the NaNO_3_ environment. Representative snapshots of the systems under study are shown in Figure 5. In addition to NaNO_3_, as shown in Figure 4b and Table 3, the cation of NH_4_Cl interacted with the phenylalanine (F) residues of Aβ_16–20_ via cation−π interactions, which might have enhanced the β-sheet formation.^42^

**Table 2:** Ratio of Each Component in the Amide I Band of the FT-IR Spectra of Aβ_16–20_ (6 mg/mL) without and with Salts (0.15 M)

	Aβ	Aβ + NaCl	Aβ + NH_4_Cl	Aβ + NaNO_3_
monomer	0.21	0.21	0.19	0.23
aggregate	0.72	0.73	0.74	0.71
β sheet	0.067	0.062	0.075	0.057

**Table 3:** Average Distances (in nm) between the Centers of Mass of the Salt Ions and Aβ_16–20_ Peptide Residues at the End of the Simulation

	NaCl		NH_4_Cl		NaNO_3_	
peptide residue	Na^+^	Cl^–^	NH_4_^+^	Cl^–^	Na^+^	NO_3_^–^
K-16	1.21 ± 0.30	0.77 ± 0.20	1.25 ± 0.32	0.76 ± 0.23	0.95 ± 0.28	0.40 ± 0.03
L-17	1.13 ± 0.29	0.93 ± 0.21	1.30 ± 0.34	1.01 ± 0.24	1.06 ± 0.22	0.67 ± 0.08
V-18	1.30 ± 0.30	1.05 ± 0.23	1.31 ± 0.33	1.11 ± 0.22	1.09 ± 0.23	0.58 ± 0.04
F-19	1.31 ± 0.30	1.02 ± 0.23	1.20 ± 0.32	1.16 ± 0.26	1.08 ± 0.26	0.88 ± 0.14
F-20	1.14 ± 0.29	0.96 ± 0.22	1.11 ± 0.35	1.06 ± 0.30	0.97 ± 0.23	0.82 ± 0.17
average	1.22 ± 0.30	0.95 ± 0.24	1.23 ± 0.34	1.02 ± 0.29	1.03 ± 0.20	0.67 ± 0.20

**Figure 6:**
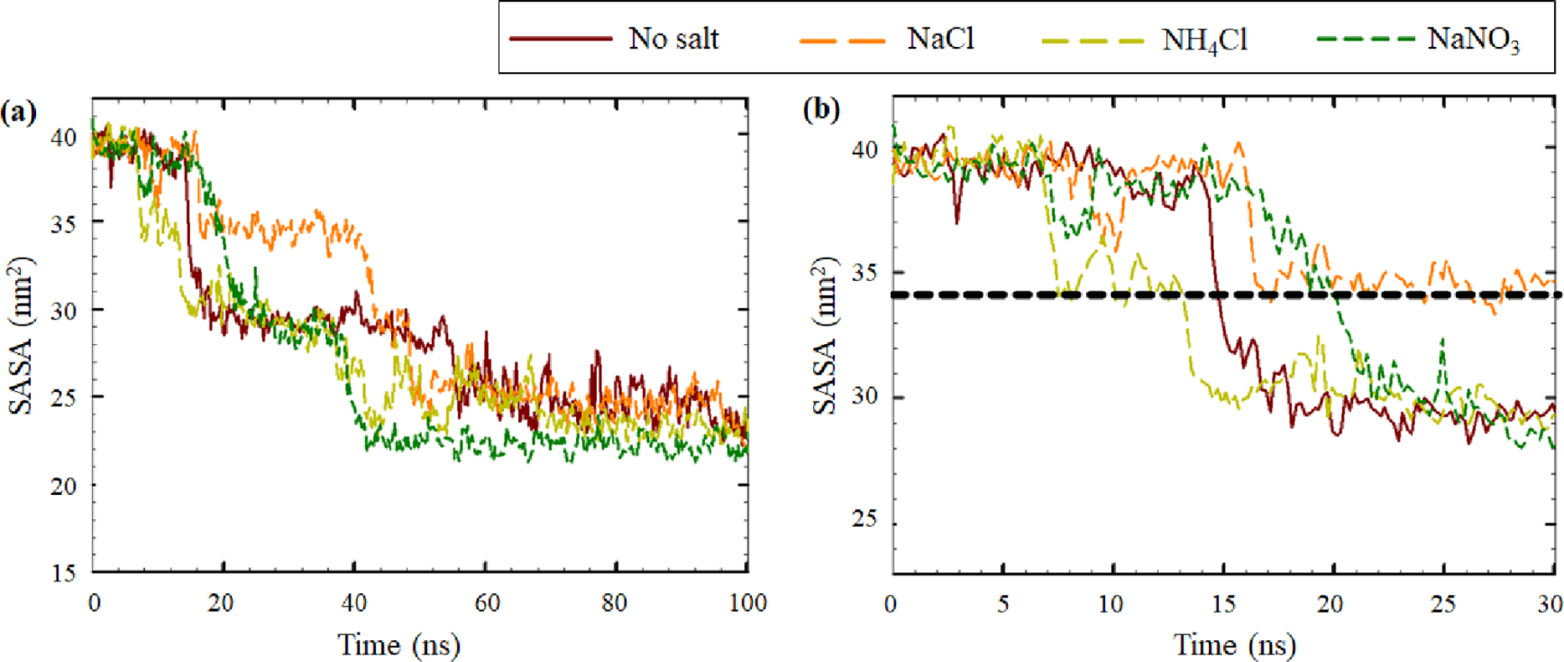
Time evolution of the total SASA of the Aβ_16–20_ peptides in the systems under study: (a) within 100 ns of the MD run and (b) within 30 ns of the MD run. The average values of each 200 ps of the simulation run were plotted, which corresponded to 50 frames of the run.

The solvent accessible surface areas (SASAs) of the peptides were further studied to compare the aggregation kinetics under different environments (Figure 6). The total SASA (SASA_0_) of the four peptides in the beginning of the simulation was 39 nm^2^, which during the 100 ns of the simulations, decreased to ∼22 nm^2^ (SASA_100_), indicating peptide aggregation (Figure 6a). The initial aggregation kinetics was quantified by estimating the time when the total SASA of peptides reached 34 nm^2^ (SASA_34_) during the first 30 ns of the simulations (Figure 6b). According to SASA plots (Figure 6b), enhanced aggregation kinetics was observed in the presence of 0.15 M NH_4_Cl (SASA_34_ was reached in 8 ns), which was related to the enhanced formation of beta sheets, observed from IR spectra (as shown previously in Table 2). The slowest aggregation kinetics was observed in the system with 0.15 M NaNO_3_ (SASA_34_ was reached in 19 ns), which corresponded to the retention of monomers in this environment, consistent with the results of IR absorption (shown previously on Table 2).

## Conclusions

We evaluated the changes in the secondary structure and assembly of the Aβ peptide (Aβ_16–20_) due to CeF_3_ NPs using liquid film FT-IR measurements and MD simulations. CeF_3_ NPs were found to locally concentrate Aβ_16–20_ on their surfaces, possibly due to the hydrophobic interaction between NPs and Aβ_16–20_ in aqueous environments, and promote the β-sheet formation of Aβ_16–20_. The concentrated Aβ_16–20_ on the NP surface formed ordered hydrogen bonds to form a β-sheet. This increase in the β-sheet formation of Aβ_16–20_ on the NP surfaces was suppressed in the presence of NH_4_^+^ and NO_3_^–^ ions. Hydrogen bonding between Aβ peptides were dominant when concentrated on CeF_3_ NP surfaces in the absence of NH_4_^+^ or NO_3_^–^. In the presence of NH_4_^+^ or NO_3_^–^, the hydrogen bonding was suppressed due to dominant bonding between the NPs and Aβ peptides. The formation of the β-sheet structure of Aβ peptides was promoted in the presence of NH_4_^+^ ions, whereas it was suppressed in the presence of NO_3_^–^ ions regardless of the presence/absence of CeF_3_ NPs, which can be explained by the electrostatic interaction between the lysine residue (amino group) of Aβ peptides and the ions. Although this study was performed using the Aβ_16–20_ peptide, future research will be conducted using full-length Aβ (Aβ_1–42_) to reveal more realistic in vivo phenomena. The analysis technique using FT-IR spectroscopy and MD will contribute to comparative studies of the effect of NPs on the molecular state of proteins under various physicochemical conditions.

## Materials and Methods

### Materials

Cerium (III) chloride heptahydrate (CeCl_3_·7H_2_O), oleic acid, deuterium oxide (D_2_O), and amyloid beta (16–20) peptide [Aβ_16–20_; Ac-Lys-(Me)Leu-Val-(Me)Phe-Phe-NH_2_] were purchased from Sigma-Aldrich Co. (St Louis, MO, USA), and 1-octadecene was purchased from Tokyo Chemical Industry Co., Ltd. (Tokyo, Japan). The N-methylated form of Aβ_16–20_ is a commercially available good model for the investigation with increased stability; however, possible changes in the peptide conformation and aggregation state associated with a possible increase in hydrophobicity should be noted. Sodium hydroxide, ammonium chloride (NH_4_Cl), sodium nitrate (NaNO_3_), sodium chloride (NaCl), methanol, ethanol, hexane, cyclohexane, and 20% deuterium chloride solution (DCl) (5.34 mol/L) in D_2_O were purchased from Fujifilm Wako Pure Chemical Co. (Osaka, Japan). Ammonium fluoride was purchased from Kanto Chemical Co., Inc. (Tokyo, Japan). All reagents were used without further purification.

### Synthesis and Characterization of CeF_3_ NPs

Fluoride NPs were synthesized via thermal decomposition.^43^ CeCl_3_·7H_2_O (1 mmol) was dissolved in distilled water (3 mL), mixed with oleic acid (12 mL) and 1-octadecene (30 mL), and stirred at 100 °C for 20 min and at 160 °C for 40 min in a nitrogen atmosphere, giving cerium oleate. After cooling to 50 °C, sodium hydroxide (2.5 mmol) and ammonium fluoride (4 mmol) dissolved in methanol were slowly added to the cerium oleate sample, and the sample was heated at 100 °C for 20 min and further at 310 °C for 50 min in a nitrogen atmosphere. The NPs collected via precipitation were purified using centrifugal washing (20 000 g, 10 min, ×3) with a hexane–ethanol mixed solvent and dispersed in cyclohexane. The NPs were characterized using XRD (Rint-Ultima 3, Rigaku Co., Tokyo, Japan) and DLS (ELSZ-2000ZS, Otsuka Electronics Co., Ltd., Osaka, Japan).

### FT-IR Spectroscopy for Samples in Solution

Fluoride NPs (27 mg/mL) in cyclohexane (750 μL) were slowly added dropwise into 1 mmol/L DCl solution in D_2_O (750 μL) and stirred for 16 h to remove cyclohexane via evaporation and to exchange the dispersion media with DCl/D_2_O. Aβ_16–20_ was dissolved in D_2_O at 8 mg/mL. The Aβ_16–20_ solution (8 mg/mL) in D_2_O with and without NH_4_Cl, NaNO_3_, or NaCl (0.2 M) was mixed with different concentrations (3–27 mg/mL) of the NP dispersion in 1 mmol/L DCl solution at a 3:1 volume ratio (thus, the final concentration of Aβ_16–20_ in the mixed samples was 6 mg/mL). The final concentrations of the NPs were set at 1–9 mg/mL because the concentration order of milligrams per milliliter is the dose commonly used for imaging contrast agents for visualizing blood flow.^27,36,44^ FT-IR spectra including amide bands were recorded using an FT/IR-6200 spectrometer (Shimadzu Co., Kyoto, Japan) for the mixed samples sandwiched between two CaF_2_ plate windows (spacer 0.025 mm). The analysis was performed for each sample within 30 min after mixing Aβ_16–20_ with the NP dispersion.

### MD Simulations

MD simulations were performed using GROMACS 2019.6 software with a GROMOS 54A7 force field. Four Aβ_16–20_ peptide monomers with a concentration of 6 mg/mL were inserted in a 9 × 9 × 9 nm^3^ box. The simulations were performed in the absence of salts and in the presence of 0.15 M NaCl, NH_4_Cl, and NaNO_3_ solutions. The MD run was performed for 100 ns for each system, following the methodology described in a previous study.^15^

### Analysis of the MD Simulations

Formation of peptide aggregates and kinetics of aggregation were studied via SASA analysis. The interactions between ions and peptide residues were studied in the last 10 ns of the simulations, when the peptide aggregates were produced. The rdf and intermolecular distance analyses were performed using the centers of mass of the peptides, averaged among four peptides. Visual molecular dynamics (VMD) software was used for the visualization of the systems under the study.
